# Supplementary material for: Glutamine supports human cytomegalovirus induced lipidome remodeling through reductive carboxylation
Source: PLoS Pathog. 2026 Aug 12;22(8):e1014496. doi: 10.1371/journal.ppat.1014496 (PMC13489524; doi:10.1371/journal.ppat.1014496)
Supplement: Supplementary file 1 [file ppat.1014496.s005.pdf]

Fig 3B

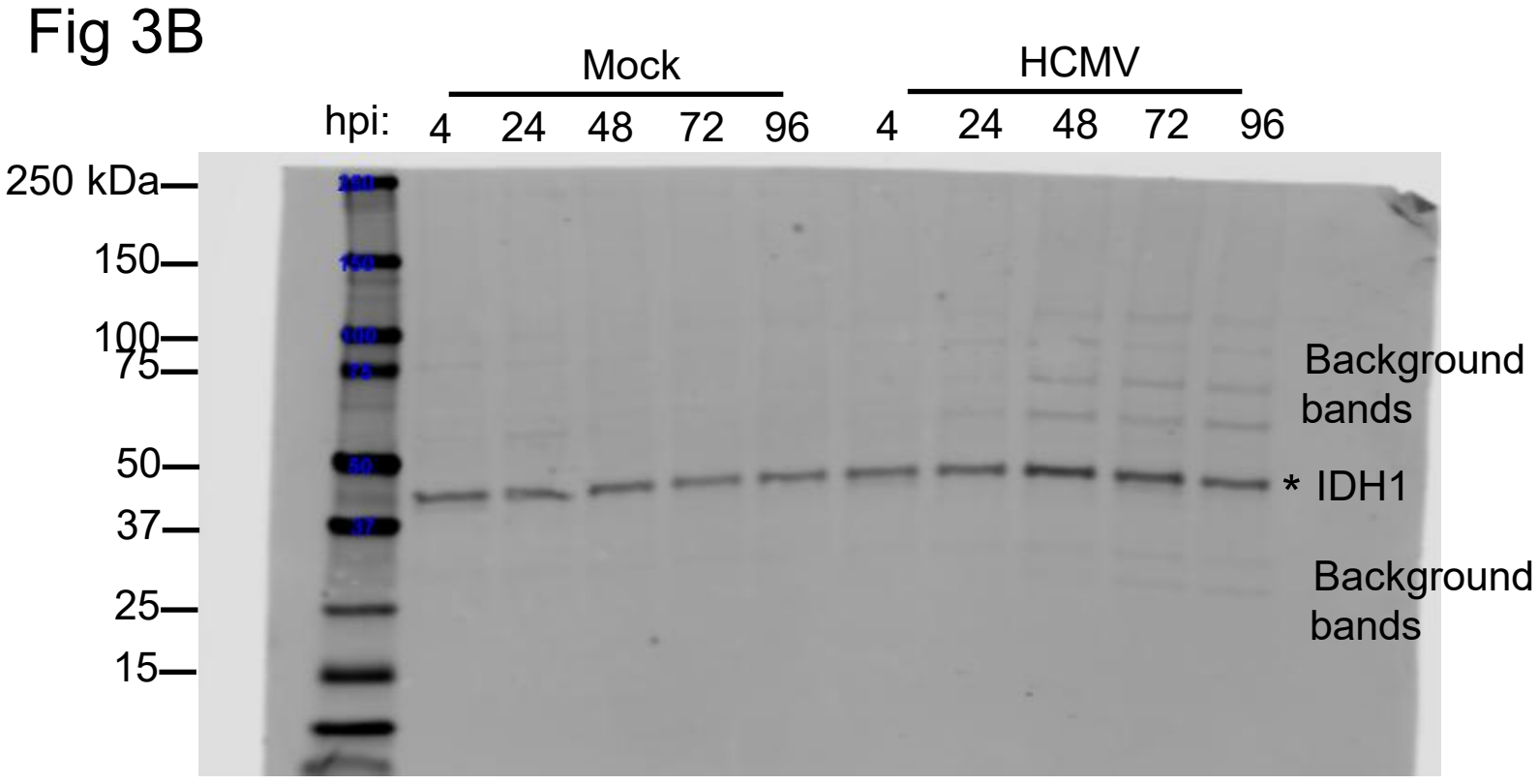

Mock
HCMV
hpi: 4 24 48 72 96 4 24 48 72 96
250 kDa
150
100
75
50
37
25
15
Background bands
* IDH1
Background bands

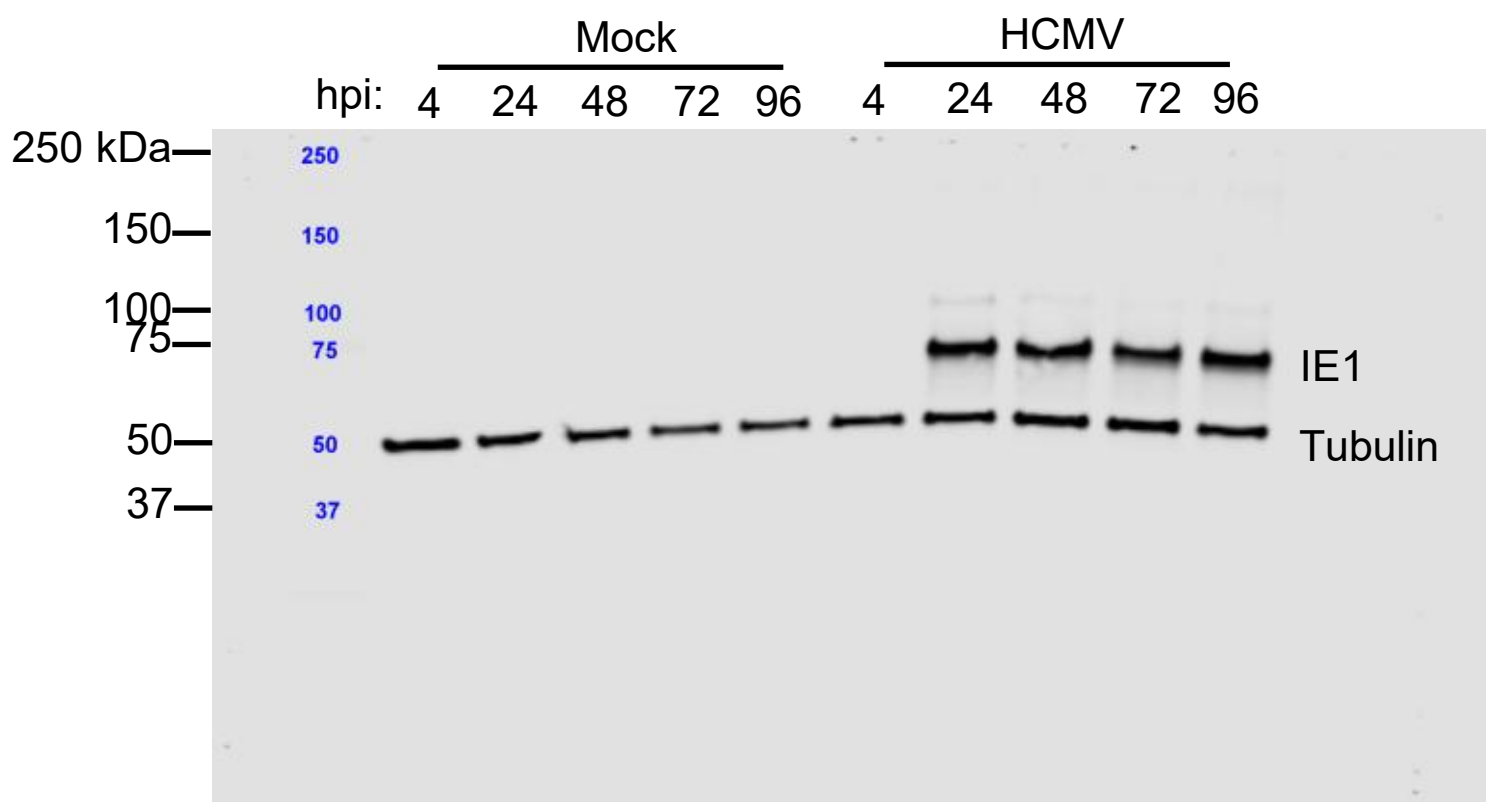

Mock
HCMV
hpi: 4 24 48 72 96 4 24 48 72 96
250 kDa
150
100
75
50
37
IE1
Tubulin

Fig 3B

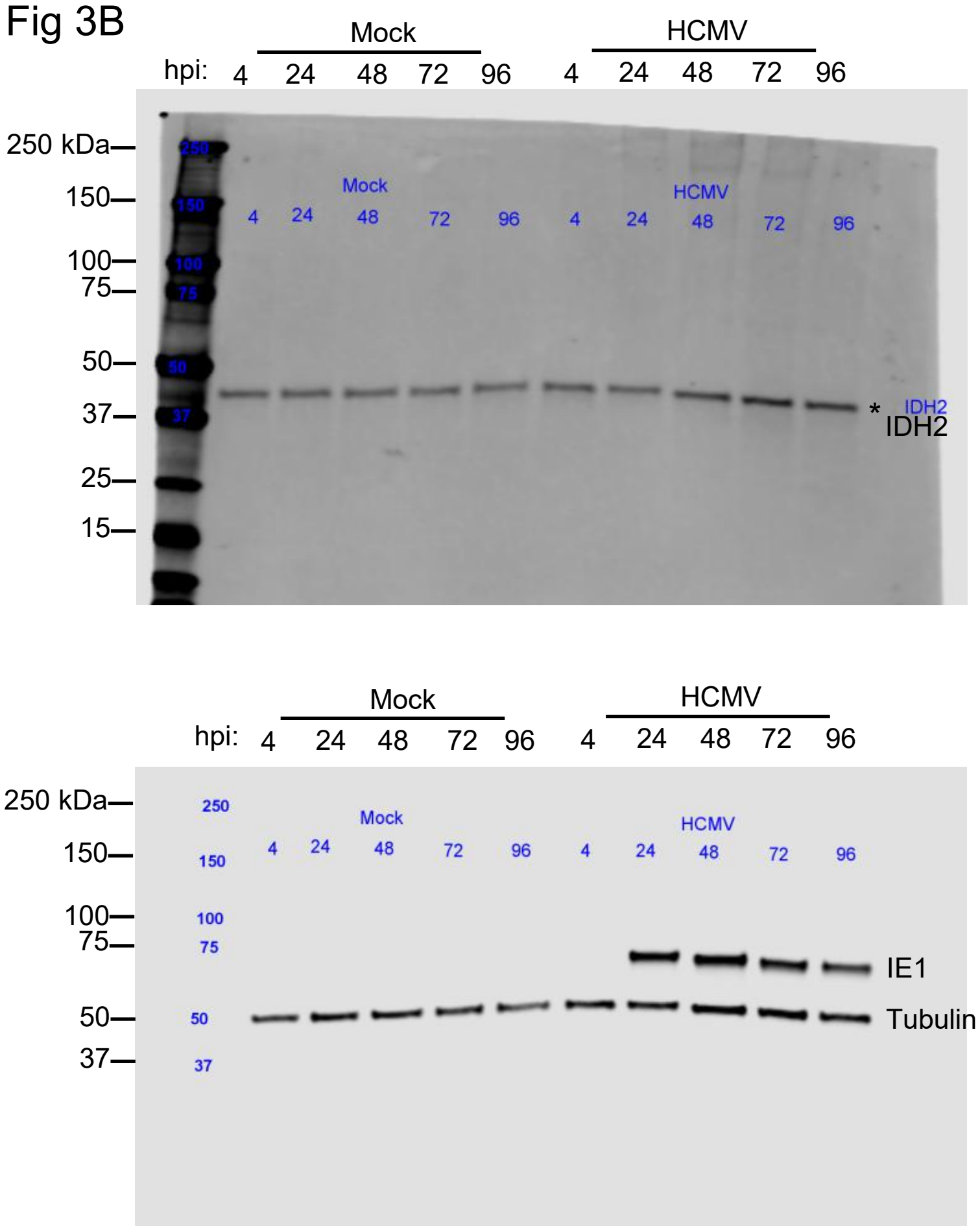

Mock
HCMV
hpi: 4 24 48 72 96 4 24 48 72 96
250 kDa
150
100
75
50
37
25
15
* IDH2
Mock
HCMV
hpi: 4 24 48 72 96 4 24 48 72 96
250 kDa
150
100
75
50
37
IE1
Tubulin

Fig 3B

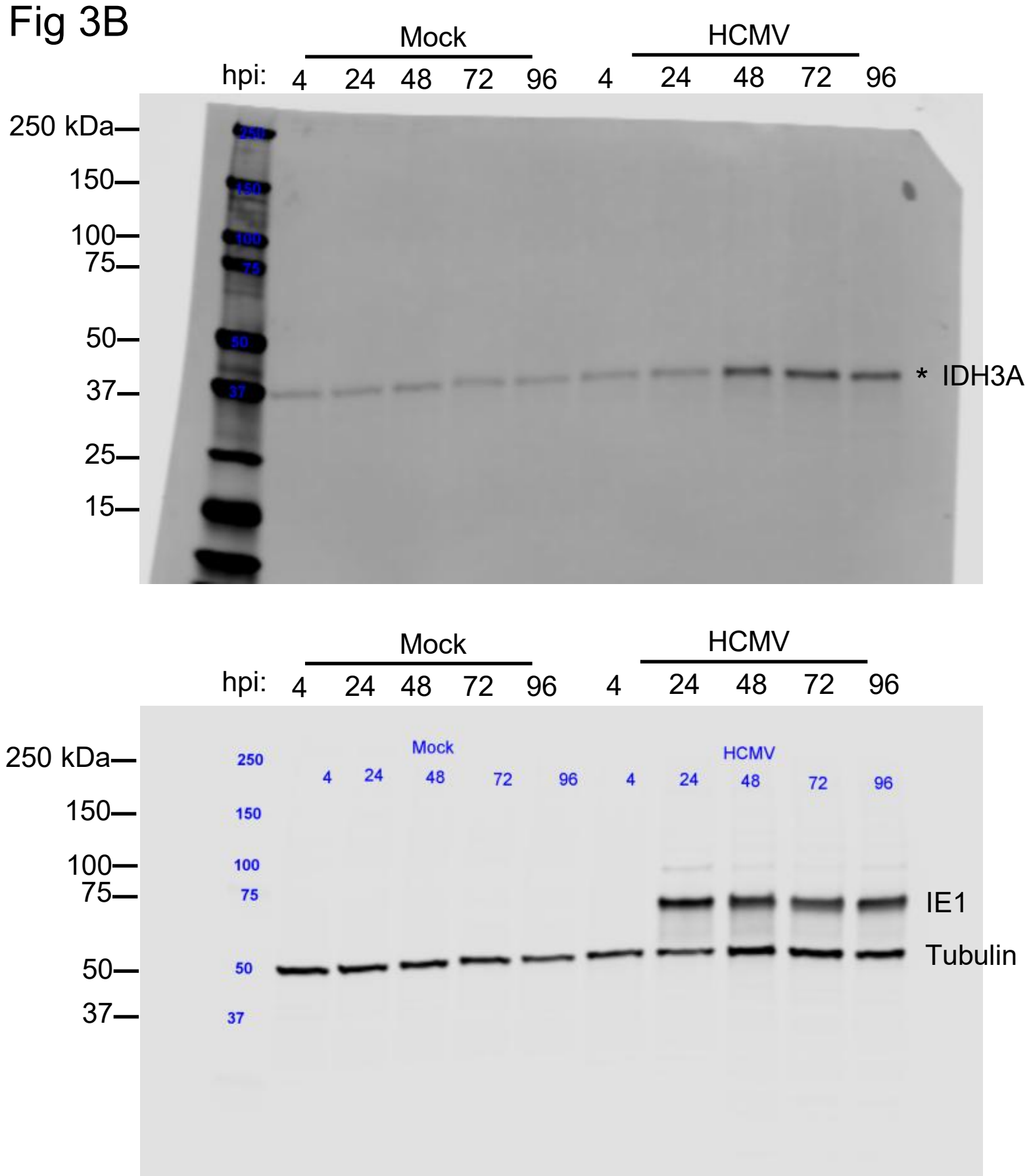

Mock
HCMV
hpi: 4 24 48 72 96 4 24 48 72 96
250 kDa
150
100
75
50
37
25
15
* IDH3A
Mock
HCMV
hpi: 4 24 48 72 96 4 24 48 72 96
250 kDa
150
100
75
50
37
IE1
Tubulin

Fig 6C

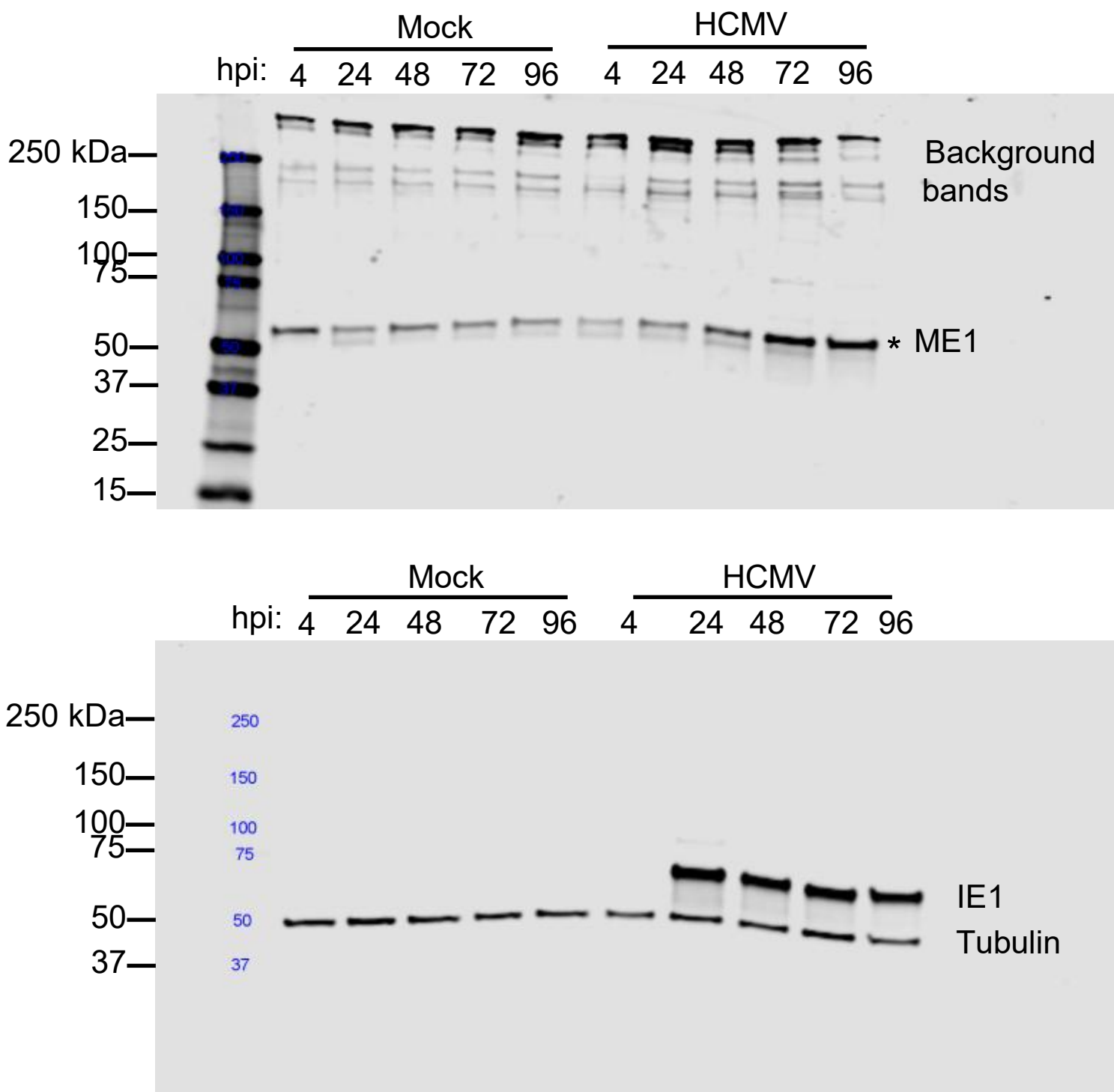

Mock
HCMV
hpi: 4 24 48 72 96 4 24 48 72 96
250 kDa
150
100
75
50
37
25
15
Background bands
* ME1
Mock
HCMV
hpi: 4 24 48 72 96 4 24 48 72 96
250 kDa
150
100
75
50
37
IE1
Tubulin

Fig 6C

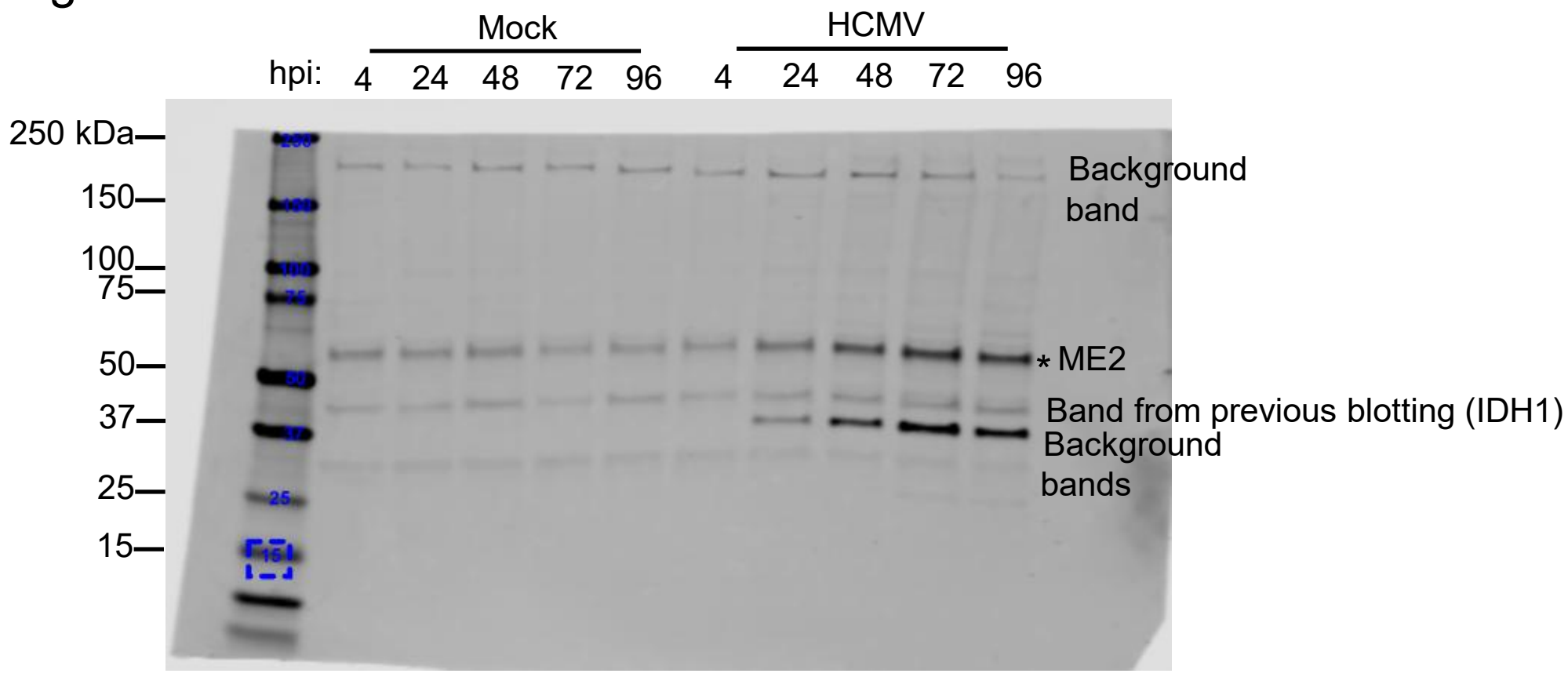

Mock
HCMV
hpi: 4 24 48 72 96 4 24 48 72 96
250 kDa
150
100
75
50
37
25
15
Background band
* ME2
Band from previous blotting (IDH1)
Background bands

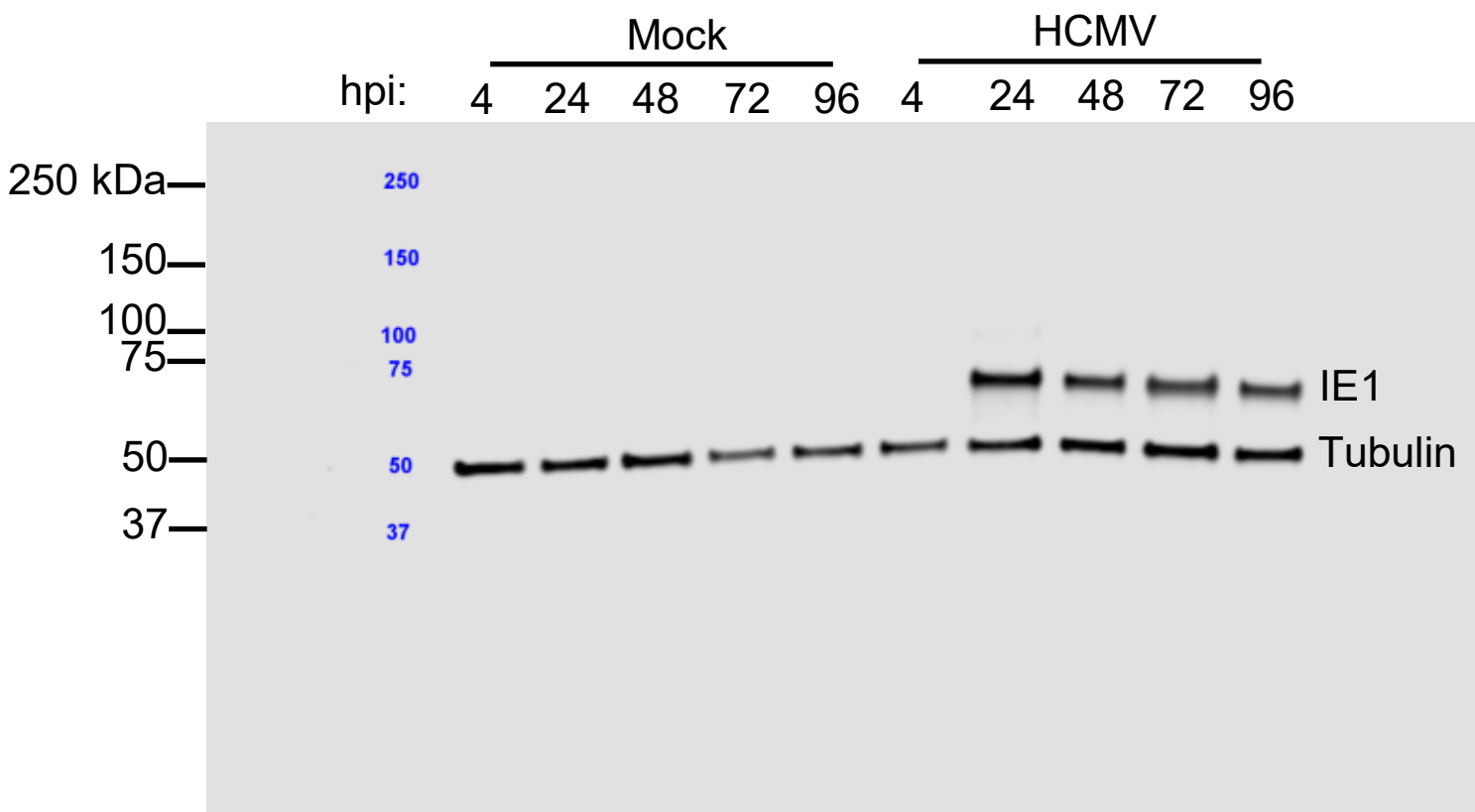

Mock
HCMV
hpi: 4 24 48 72 96 4 24 48 72 96
250 kDa
150
100
75
50
37
IE1
Tubulin

Fig 6C

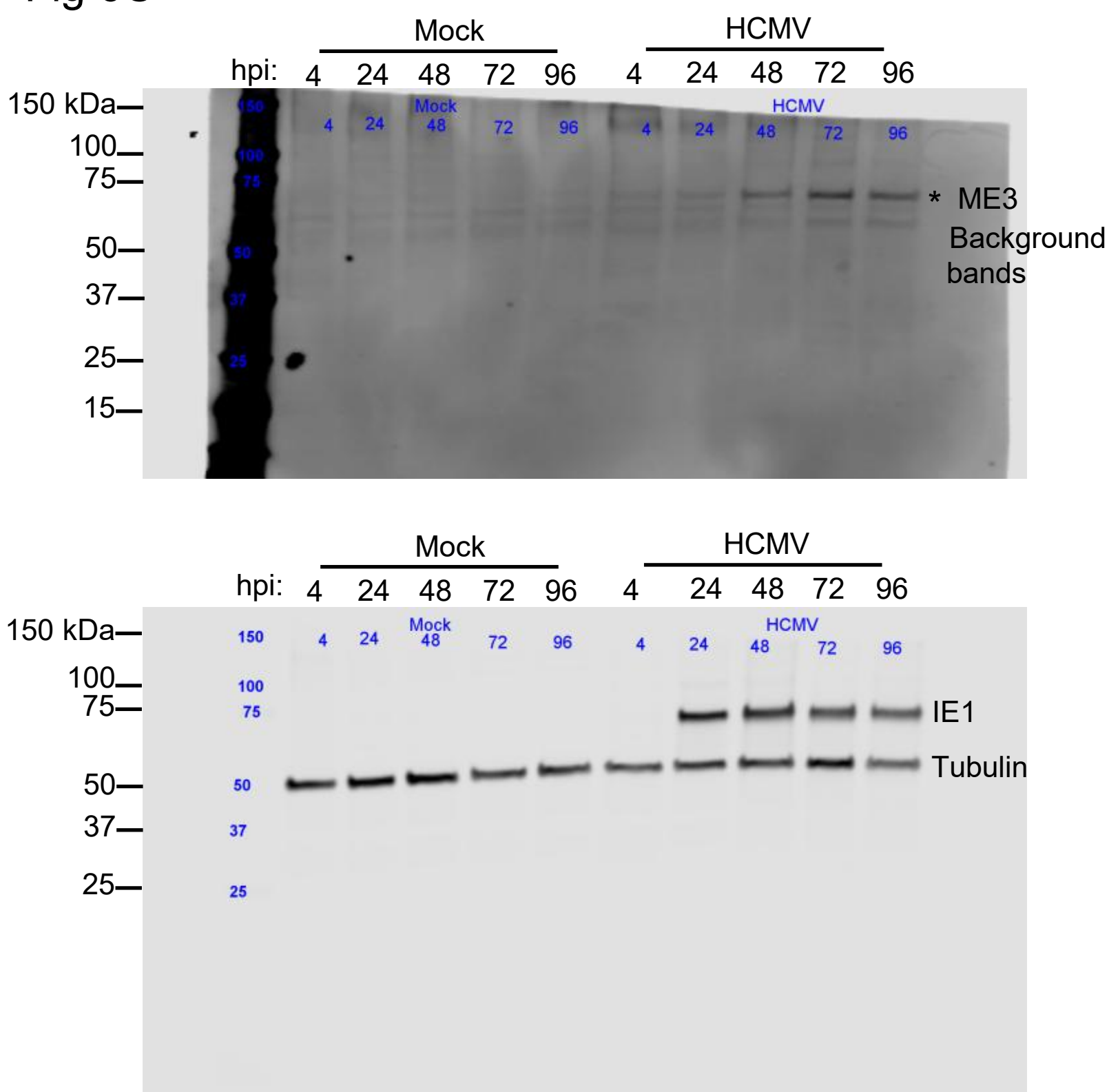

Mock
HCMV
hpi: 4 24 48 72 96 4 24 48 72 96
150 kDa
100
75
50
37
25
15
* ME3
Background bands
Mock
HCMV
hpi: 4 24 48 72 96 4 24 48 72 96
150 kDa
100
75
50
37
25
IE1
Tubulin

Fig 7G

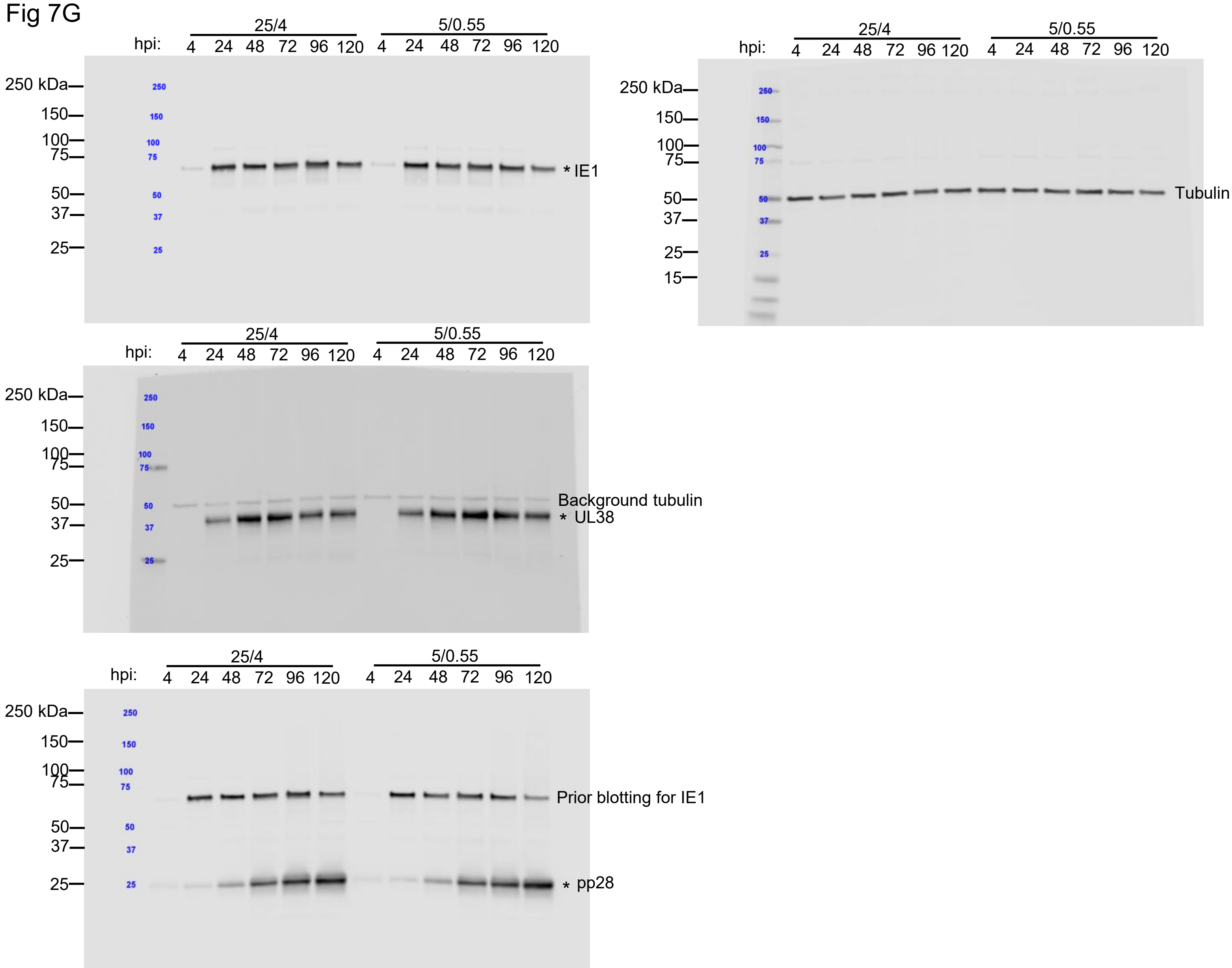
25/4
5/0.55
hpi:
4 24 48 72 96 120 4 24 48 72 96 120
250 kDa
150
100
75
50
37
25
15
* IE1
Tubulin
Background tubulin
* UL38
Prior blotting for IE1
* pp28

Fig S4

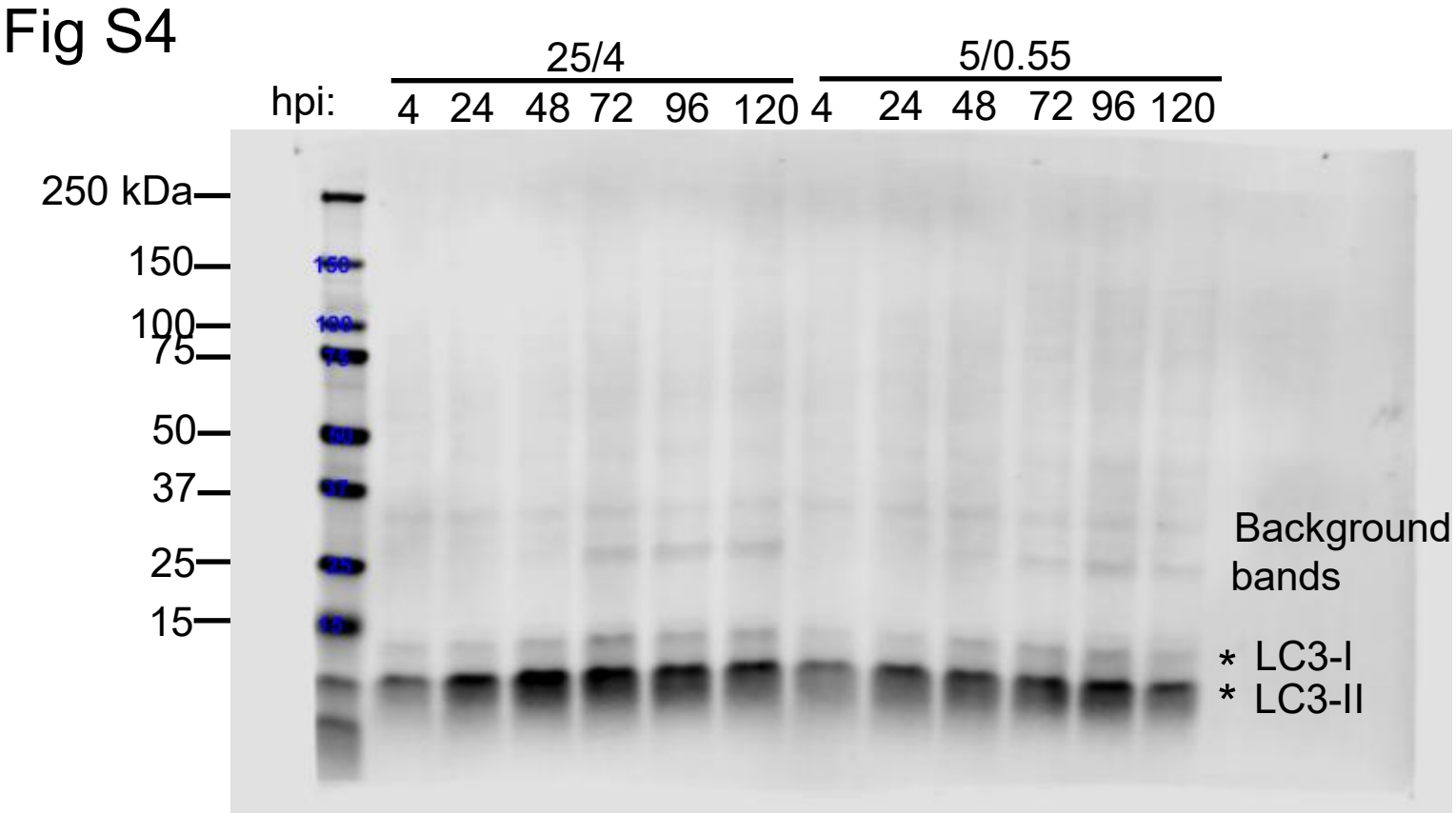

25/4
5/0.55
hpi:
4 24 48 72 96 120 4 24 48 72 96 120
250 kDa
150
100
75
50
37
25
15
Background bands
* LC3-I
* LC3-II

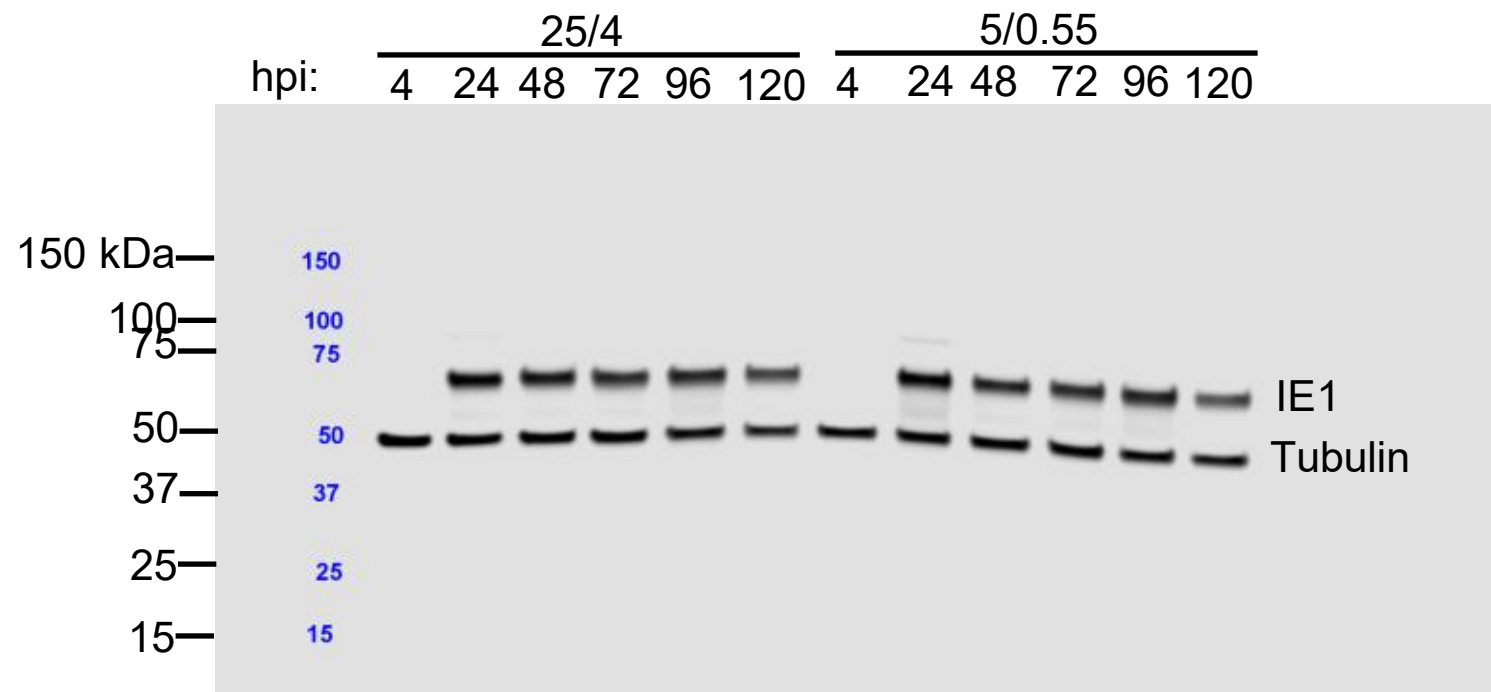

25/4
5/0.55
hpi:
4 24 48 72 96 120 4 24 48 72 96 120
150 kDa
100
75
50
37
25
15
IE1
Tubulin
