# Supplementary material for: Glutamine supports human cytomegalovirus induced lipidome remodeling through reductive carboxylation
Source: PLoS Pathog. 2026 Aug 12;22(8):e1014496. doi: 10.1371/journal.ppat.1014496 (PMC13489524; doi:10.1371/journal.ppat.1014496)
Supplement: Supplementary file 2 [file ppat.1014496.s007.docx]

Dear Editor,

We are grateful for the reviewers’ feedback and helping us improve the manuscript. In response to the reviewers’ input, we performed additional experiments and provided further clarifications. Additionally, we appreciate PLoS Path’s commitment to a rigorous and timely review process. Thank you for moving our manuscript forward in the review process and allowing us to resubmit our manuscript. A point-by-point response is detailed below. The *reviewers’ comments are italicized in blue,* and our responses are in black.

Thank you,

John Purdy

**Reviewer #1:**

**Summary:** *Overall, the authors’ conclusions are nicely supported by the data. The manuscript is also well-written, and the authors provide helpful schematics throughout to allow the reader to understand the metabolic pathway at-hand. Finally, the overall model presented in figure 9 nicely shows the difference between what we knew (panel A) and what this study now reveals (panel B).*

Thank you for the thoughtful consideration of our study. The feedback provided was very much appreciated.

**Minor Issue 1.** *Define abbreviations at first use (e.g., line 124 – PC, PE, etc).*

Thank you for your attention to these details. We have defined abbreviations. The updated manuscript now states:

Lines 121-127: “*We also examined lysophosphatidylcholine (LPC) and lysophosphatidylethanolamine (LPE) that contain one fatty acyl tail since interconversion between one-tailed LPC or LPE with two-tailed PC or PE is active in fibroblasts (9, 30). By 48 hpi, the percentage of phosphatidylcholine (PC), phosphatidylethanolamine (PE), phosphatidylinositol (PI), diacylglycerol (DG), and triacylglycerol (TG) labeled carbons were increased in HCMV-infected cells compared to mock (****Fig 1A****), and by 72 hpi, these lipids had more pronounced labeling (****Fig 1B****).”*

**Minor Issue 2.** *Paragraph ending on line 135 – perhaps a brief phrase detailing what no alteration in PG might suggest is warranted.*

Thank you for this prompt. We added to the results and discussion sections to expand on this observation.

Lines 136-138: *“Phosphatidylglycerol (PG) was the only lipid class that we examined where infection did not alter labeling from glutamine, suggesting that HCMV does not promote PG synthesis using glutamine carbons (****Fig 1A-B****).”*

Lines 490-496: *“PG is primarily synthesized in the mitochondria unlike other phospholipids that are synthesized at the endoplasmic reticulum (55). Some PG with long-chain fatty acid tails with two or fewer double-bonds are enriched in HCMV-infected cells, while most others are unchanged or reduced (7, 8). Further research is needed to understand the role of PG in HCMV-infected cells and the impact infection has on their metabolism. Nonetheless, our data show that HCMV infection does not promote the contribution of glutamine to the synthesis of PG lipids.”*

**Minor Issue 3.** *Line 148 – re: diunsaturated. This looks to only be the case for PC and not PE? Perhaps clarify?*

Thank you for this observation regarding the lipids that have the greatest levels of ^13^C-glutamine enrichment. We agree the wording was unclear. We have added additional details regarding the description of diunsaturated PC and PE.

Line 148-158: “*We focused on lipid classes with the highest levels of labeling from glutamine in HCMV-infected cells compared to mock, i.e., PC, PE, and DG. For labeled PC and PE, HCMV significantly increased the percent ^13^C in all tail types, with greatest enrichment for saturated and monounsaturated (****Figs 1C-D*** *and* ***S1A-B****). For labeled PC in HCMV-infected cells, we observed that most diunsaturated tail types had a 2-fold or greater level of labeling, while a small cluster had a lower level of labeling (****Fig 1C****). This distribution was the result of an enrichment in labeled diunsaturated PC species as the carbon chain length increased, e.g., PC(48:2) had a greater level of labeling than PC(34:2) (****Fig S1A****). Although less robust than diunsaturated PC, diunsaturated PE labeling was also enriched in HCMV-infected cells, including a higher level of labeling in the longest PE species we measured, PE(44:2) (****Fig 1D and S1B****).”*

**Minor Issue 4.** *Figure 7, figure legend – define LOQ.*

We apologize for the oversight. We have updated Figure 7 and since all data points are well above the limit of quantification (LOQ) of the assay, we removed “LOQ” from the figure.

**Minor Issue 5.** *TCID50 data in Figure 7 – what were the media conditions used for the titering assay? Were both the 25/4 and 5/0.55 conditions being assessed normalized into one media for the TCID50 assay, or were they each retained in their respective media for titering? Could this impact the output?*

Thank you for your question; we apologize for our oversight in describing this in the first submission. Both sets of conditions were titered in standard DMEM which contains 25mM glucose and 4 mM glutamine with 10% FBS. By normalizing the conditions for TCID_50_, we are able to assess only infectious virus production without confounding factors that may happen if the titer assay conditions differ.

We have added information on media conditions for TCID_50_ assays to the methods section.

Lines 579-582: *“All TCID50 assays were completed in DMEM (Gibco 11965) containing 25 mM glucose and 4 mM glutamine supplemented with 10% fetal bovine serum (FBS), 10 mM HEPES, and penicillin/streptomycin (P/S).”*

**Minor Issue 6**. *Lines 340-1 – since this decrease occurs in mock cells, could this also be a reason for the decrease observed in infected cells between the two conditions (ie: a cell phenotype vs. virus phenotype)?*

Glucose labeling of FA was reduced in mock-infected cells in physiological glucose and glutamine (**Fig 8D-E**). In contrast, in HCMV-infected cells we observed equal levels of ^13^C-glucose labeling in standard DMEM and DMEM with physiological levels of glucose and glutamine (**Fig. 8D-E**). This demonstrates that infection continues to promote FA synthesis in physiological glucose and glutamine conditions.

Glutamine labeling tells a different story. Glutamine labeling of FA was reduced in both mock and HCMV-infected cells in physiological glutamine concentrations (**Fig 8A-B**). Thank you for asking this question. Upon reflection, we agree that these data suggest reduced ^13^C-glutamine labeling in physiological glucose and glutamine conditions is likely cell-mediated. We have now included the following in the Discussion.

Lines 529-537: “*Physiological glucose and glutamine levels did not alter increased FA abundance during HCMV infection but did impact carbon suppliers for FA synthesis. Glucose labeling of FA was reduced in mock-infected cells, but HCMV infection continues to promote the flow of carbons from glucose to FA synthesis (****Fig 8D-E****). In contrast, glutamine labeling of FA was reduced in both mock and HCMV-infected cells in physiological glucose and glutamine concentrations (****Fig 8A-B).*** *This observation suggests that cells reduce the flow of glutamine carbons to FA synthesis in response to the lower concentrations of glucose and glutamine. Further, this result suggests that while HCMV infection increases the flow of glutamine to FA, infection cannot completely overcome the impact that reduced glucose and glutamine have on the cell.”*

**Minor Issue 7.** *Line 370 – extra word before HCMV? (remove ‘the’?)*

Thank you. We have removed the extra word.

**Reviewer #2:**

**Summary:** *The manuscript is very well written, and the lipidomics and tracer experiments are methodologically rigorous. Further, the experiments support their conclusions.*

We thank the reviewer for acknowledging the strengths of our work.

*However, reductive flow of glutamine to lipids is a widely accepted cellular metabolic activity, with papers describing it over 15 years ago (PMID: 18364355), with subsequent confirmation in many contexts at the time (PMID: 22101433; 22101431). These findings limit the importance of describing these phenotypes in infected cells. The manuscript does not provide new insights into how HCMV might directly modulate specific metabolic activities, nor do they examine how specific metabolic activities might contribute to productive infection. They speculate about contributions that malic enzyme or IDH might be making, but they never test their role. As it stands, their findings are not likely to be of broad general interest and are likely more suitable for a more specialized journal.*

We appreciate the feedback regarding the relevance of our findings. Very little is known about reductive carboxylation during virus infection, despite the wide acceptance of this metabolic pathway. Many viruses are known to require glutamine for replication, but only two studies have described active reductive carboxylation in mammalian viruses -adenovirus type 5 (PMID: 26561297) and murine norovirus (PMID: 38976719). Both papers focused on glutaminolysis activity and identified active reductive carboxylation through measuring 3-labeled intermediates using ^13^C-glutamine tracing. HCMV is known to require glutamine for replication, but its role in replication is incompletely understood. Importantly, prior to our study, it was only thought to support virus replication through anaplerosis. Our study adds new insight into the role of glutamine in lipid synthesis during virus infection. We demonstrate that reductive carboxylation is directly responsible for the majority of glutamine carbon flow to fatty acid and lipid synthesis during virus infection. Moreover, we provide additional novel insight into this process during virus infection by comparing labeling from different forms of glutamine to demonstrate that reductive carboxylation is not the only pathway by which glutamine can contribute carbons to FA synthesis. These findings are novel.

Given that our work advances our understanding of glutamine utilization, the labeling of lipids, and the discovery that carbons enter beyond just reductive carboxylation to citrate, we feel the work provides a complete story that answers new questions and generates new hypotheses. Our discussion of malic enzyme pathway is an example of a new hypothesis that warrants further investigation based on our current findings. Having additional new hypotheses to pursue is exciting and important. Overall, our study provides targets for future studies to understand the virus-host interaction in novel ways.

In response to this comment, we provided edits the discussion to place our story in broader context to emphasize the unique findings from our research.

Lines 429-446: *“Reductive carboxylation is widely recognized as the primary pathway by which glutamine contributes to FA and lipid synthesis (18, 31, 45, 46). However, its contribution to the pro-viral metabolic environment is only beginning to be interrogated. Our data demonstrate that HCMV promotes reductive carboxylation during replication (****Figs 4****,* ***5****,* ***9****,* *and* ***S3****). In the context of virus infection, reductive carboxylation activity has been reported in only four other viruses, including two other mammalian virus infections: Adenovirus type 5 (Ad5) (non-enveloped, DNA virus) and murine norovirus (MNV) (non-enveloped, RNA virus) infection (47-50). These studies identified an increase in 3-labeled TCA cycle metabolites during infection after culture in media containing U-^13^C-Gln. Malate, fumarate, or aspartate with 3-labeled carbons are only obtained through reductive carboxylation of U-^13^C-Gln after citrate cleavage to acetyl-CoA and oxaloacetate. These studies did not directly measure glutamine carbon incorporation into FA and lipids. Our data establish that reductive carboxylation supports FA synthesis during virus replication and remains the primary pathway by which glutamine contributes to FA synthesis (****Figs 2, 4****,* ***5****,* ***9****,* ***S2****,* *and* ***S3****). Moreover, by comparing FA labeling from uniformly and positionally labeled ^13^C-glutamine tracers, we demonstrated that additional carbons from glutamine are used for FA synthesis (****Fig 5****). Our results from HCMV, coupled with studies on Ad5 and MNV, indicate that reductive carboxylation is likely a shared metabolic mechanism in a diverse range of enveloped and non-enveloped DNA and RNA viruses.”*

**Major issue 1.** *Is malic enzyme important for HCMV-induced lipid biosynthesis or viral infection? What about the activities of IDH isoforms, e.g., IDH1 or IDH2? The authors propose these possibilities, but don’t test the possible contributions to lipid biosynthesis or viral infection.*

Thank you for your question. We are actively working on follow-up studies focused on malic enzyme contribution to lipid metabolism and productive HCMV replication. So far, we have knocked down malic enzyme 1 (ME1). We are also studying isocitrate dehydrogenases (IDH). We measured virus production in IDH1 knockout (KO) cells. Our preliminary findings show that IDH1 and ME1 are not required for HCMV production in standard DMEM conditions (see figure below). Moreover, we found FA abundance in this condition is still increased during HCMV infection in ME1-KD cells. We have not yet performed isotope tracing in ME1-KD cells. We are currently investigating the role of ME1 in different metabolic environments. Surprisingly, we found that ME1 is required for cell viability during glucose deprivation in HCMV-infected cells, an observation we are continuing to investigate. We are continuing this work by examining other metabolic environments and investigating the cell viability requirement of ME1 in glucose-free conditions. Moreover, we are in the process of knocking down or out ME2-3 and IDH2-3 to study their roles in HCMV infection in several metabolic environments as well. Since these additional investigations represent a large amount of work that will take many months and will likely result in their own novel findings, we are including our preliminary data in this response document to the reviewers, but the research will be published later once the studies are completed. These future studies are built on the foundations established in the current manuscript under consideration. Importantly, these ongoing and future follow-up studies are not required to draw the novel conclusion that glutamine contributes to FA and lipid synthesis via reductive carboxylation during virus infection and an additional pathway, malic enzyme pathway, may also contribute.

**Major issue 2.** *It is not clear whether HCMV infection is increasing the reductive to oxidative ratio of glutamine-carbon into lipid species relative to mock infection. While the authors find increased 13C-glutamine labeling of lipids/fatty acids, given that HCMV dramatically induces lipogenesis during infection, the increased tracer labeling could reflect this increased lipid biosynthesis and not necessarily a change in the oxidative/reductive processing of glutamine carbon (via aKG).*

Thank you for this observation. We agree that our data provides no evidence that glutamine is preferentially shuttled to oxidative TCA cycle or reductive carboxylation. This is an important idea to address as we seek to understand the role of glutamine in HCMV replication and how infection regulates its use. This is an aspect of the project that the lead author, Dr. Mokry, plans to continue to study when she establishes her own lab.

Nonetheless, we updated our manuscript to ensure our wording doesn’t lead to confusion or overstate the conclusions that can be drawn from our current work.

Lines 406-428: *”Our previous understanding of glutamine during HCMV replication was to maintain the TCA cycle through glutaminolysis, i.e., catabolism of glutamine to α-ketoglutarate that feeds the TCA cycle (6, 16). In addition to its role in TCA cycle maintenance, our data demonstrate that glutamine is also metabolized through reductive carboxylation to support lipid synthesis (****Fig 9****). Together, these results indicate that HCMV promotes both oxidative TCA cycle and reductive carboxylation during replication. Our data cannot elucidate the ratio of glutamine contributing to oxidative TCA cycle versus reductive carboxylation. Both metabolic uses of glutamine may be essential for HCMV replication. Glutamine deprivation inhibits HCMV replication but starves both oxidative TCA cycle and reductive carboxylation of glutamine in infected cells (16, 17). Chambers et al. demonstrated that α-ketoglutarate, oxaloacetate, and pyruvate supplementation are each sufficient to restore virus production during glutamine deprivation (16). Each supplement supports both metabolic pathways. α-Ketoglutarate can feed reductive carboxylation and oxidative TCA cycle, while oxaloacetate is a product of both pathways via cytosolic citrate cleavage (****Fig 9****). Pyruvate can be produced from glucose through glycolysis or from glutamine via the malic enzyme pathway (****Figs 5-6****) and can also be converted into oxaloacetate via pyruvate carboxylase if it is active. One possibility is that the virus has evolved to promote glutaminolysis that supports either oxidative TCA cycle or FA synthesis in specific metabolic niches where nutrients may be limiting (16). It remains to be investigated how these two processes are regulated, and if differing nutrient environments alter their regulation and contribution to the metabolic needs of HCMV-infected cells. Although our study and the study by Chambers et al. cannot elucidate the essentiality of oxidative TCA cycle versus reductive carboxylation, they highlight the remarkable metabolic adaptability occurring in HCMV-infected cells.*”

**Major issue 3**. *They saw a strong reduction in viral titers when comparing rich media to the media with 5 mM glucose & 0.55 mM glutamine (Fig 7F). They indicate that they change the media at 48h post-infection. Given the more than 100-fold effect observed, it raises the question as to whether the media concentration of glucose and glutamine drops precipitously, maybe at 72-120 h post-infection, causing a starvation/autophagic type of response. This is also relevant to whether media concentrations stay at physiologically relevant concentrations, i.e., as opposed to dropping significantly lower.*

We agree with the reviewer that this is a major caveat that happens in our cell culture model. In response to this critique and minor issue 1, we completed additional experimentation. We had previously quantified MOI 1 and MOI 3 infections at one time (120 hpi). In response to this comment, we repeated the 120 hpi and added a 144 hpi collection which would represent an even further drop in metabolite concentrations in the media. Further, we also measured viral protein production over a time course of infection. If metabolites necessary for viral protein synthesis were depleted, we would expect a reduction in viral proteins. In a third experiment in response to this comment, we examined autophagy markers.

The new findings are:

1. Virus titers at 120 hpi are similar to 144 hpi in the medium with reduced glucose and glutamine levels (5/0.55). New data shown in **Fig 7F** (provided below for your convenience).
2. Viral protein levels are similar in standard DMEM and 5/0.55 DMEM. New data shown in **Fig 7G-J** (provided below for your convenience).
3. Autophagy markers LC3-I and LC3-II are similar in standard DMEM and 5/0.55 DMEM. New data shown in **Fig S4** (provided below for your convenience).

While these new results do not directly measure the concentration of glucose and glutamine following 48 hpi, they demonstrate that the level of glucose and glutamine are sufficient enough to support major viral replication steps until 144 hpi. Moreover, our observations suggest that these cells cultured in 5/0.55 DMEM are not altering the autophagic response during HCMV replication.

Lines 327-339: ***“****Given this finding, we measured viral protein levels from the canonical kinetic classes of HCMV lytic replication. We found no difference in viral protein levels of any kinetic class, i.e., immediate-early, early, or late (****Fig 7G-J****). We next asked if reduced HCMV production was the result of altered autophagic activation in 5/0.55 media. HCMV hijacks autophagic machinery for secondary envelopment and particle maturation (42), while nutritional stress activates autophagy (43). We reasoned that nutrient stress from reduced levels of glucose and glutamine in 5/0.55 conditions could alter the autophagic response in HCMV-infected cells. We compared the levels of a common marker of autophagosomes, LC3-I and its lipidated form LC3-II, and found no difference in 5/0.55 DMEM conditions (****Fig S4****). Overall, these results demonstrate that despite reduced infectious virus production, physiological glucose and glutamine levels are sufficient to support increased FA abundance and viral protein production that occurs during HCMV replication in supraphysiological glucose and glutamine conditions.*”

Lines 523-528: **“***Since viral protein levels were similar in both media conditions (****Fig 7G-J****), we investigated if autophagy was altered by the media with reduced glucose and glutamine. Previous studies showed that HCMV infection induces autophagy, including increasing the levels of LC3-I and LC3-II autophagosome markers (57-60).We found similar levels of LC3-I and LC3-II in HCMV-infected cells in both standard DMEM and 5/0.55 DMEM (****Fig S4****), indicating that the defect in HCMV production is not due to an altered autophagic response.*”

**New Figure 4F-J**

**New figure S4: LC3I-II levels in physiological glucose and glutamine**

**Minor issue 1.** *In Fig 4A, in the schematic, it’s probably worth showing how the labeled 5’ position of glutamine is lost during oxidative TCA cycling.*

Thank you for the suggestion. We added a schematic showing the 5’ positional label will not label FA through oxidative turning of the TCA cycle. The new schematic is shown in **Figure 4B.**

Lines 224-228: ***“****Metabolism of 5-^13^C-Gln through oxidative TCA cycle yields 1-labeled α-ketoglutarate that upon oxidative turning of the TCA cycle will result in unlabeled acetyl-CoA, since the labeled carbon is retained in OAA following the conversion of citrate to acetyl-CoA and OAA. If the acetyl-CoA produced through this pathway were to be used for FA synthesis, it would result in unlabeled FAs (****Fig 4B****).*”

**Minor issue 2**. *It seems strange that they see 106 viral titers at MOI=1 but 104 at MOI=3.0 (Fig 7F &7G). Is this MOI dependence in replication real/repeatable?*

Thank you for this observation. As described above in response to major issue 3, we have repeated this experiment at 120 and 144 hpi, again using MOI 1 and MOI 3. The new data is shown in **Figure 7F** (and above).
