# Supplementary material for: Glutamine supports human cytomegalovirus induced lipidome remodeling through reductive carboxylation
Source: PLoS Pathog. 2026 Aug 12;22(8):e1014496. doi: 10.1371/journal.ppat.1014496 (PMC13489524; doi:10.1371/journal.ppat.1014496)
Supplement: Peer review history

# Peer review: Decision Letter 0

**DOI:** 10.1371/journal.ppat.1014496.r001

**Authors:** Eain Murphy

7 May 2026

PPATHOGENS-D-26-00709

Glutamine supports human cytomegalovirus induced lipidome remodeling through reductive carboxylation

PLOS Pathogens

Dear Dr. Purdy,

Thank you for submitting your manuscript to PLOS Pathogens. After careful consideration, we feel that it has merit but does not fully meet PLOS Pathogens's publication criteria as it currently stands. Therefore, we invite you to submit a revised version of the manuscript that addresses the points raised during the review process.

Please submit your revised manuscript by Jul 06 2026 11:59PM. If you will need more time than this to complete your revisions, please reply to this message or contact the journal office at plospathogens@plos.org. When you're ready to submit your revision, log on to https://www.editorialmanager.com/ppathogens/ and select the 'Submissions Needing Revision' folder to locate your manuscript file.

Please include the following items when submitting your revised manuscript:

* A letter that responds to each point raised by the editor and reviewer(s). You should upload this letter as a separate file labeled 'Response to Reviewers'. This file does not need to include responses to any formatting updates and technical items listed in the 'Journal Requirements' section below.

* A marked-up copy of your manuscript that highlights changes made to the original version. You should upload this as a separate file labeled 'Revised Manuscript with Track Changes'.

* An unmarked version of your revised paper without tracked changes. You should upload this as a separate file labeled 'Manuscript'.

If you would like to make changes to your financial disclosure, competing interests statement, or data availability statement, please make these updates within the submission form at the time of resubmission. Guidelines for resubmitting your figure files are available below the reviewer comments at the end of this letter.

As the corresponding author, your ORCID iD is verified in the submission system and will appear in the published article. PLOS supports the use of ORCID, and we encourage all coauthors to register for an ORCID iD and use it as well. Please encourage your coauthors to verify their ORCID iD within the submission system before final acceptance, as unverified ORCID iDs will not appear in the published article. Only the individual author can complete the verification step; PLOS staff cannot verify ORCID iDs on behalf of authors.

We look forward to receiving your revised manuscript.

Kind regards,

Eain A Murphy, Ph.D.

Academic Editor

PLOS Pathogens

Alison McBride

Section Editor

PLOS Pathogens

Sumita Bhaduri-McIntosh

Editor-in-Chief

PLOS Pathogens

orcid.org/0000-0003-2946-9497

Michael Malim

Editor-in-Chief

PLOS Pathogens

orcid.org/0000-0002-7699-2064

**Additional Editor Comments :**

Dear Dr. Purdy,

Let me begin by apologizing for the extended amount of time in your manuscript's review process. One reviewer failed to return a review after three weeks. However, your manuscript has been reviewed by two experts in the field of herpesvirology with a distinct focus on metabolic activity of infected cells. Both reviewers were of the opinion that this work is well presented, of importance and that the presented data supports the conclusions. Reviewer #2 had some concerns that I believe you can address in a resubmission. In light of this, the editorial staff has come to a conclusion of Minor Modify for this work. We look forward to receiving your resubmission for evaluation.

Cheers,

Eain Murphy

**Journal Requirements:**

1) Please ensure that the CRediT author contributions listed for every co-author are completed accurately and in full.

At this stage, the following Authors/Authors require contributions: Rebekah L Mokry, Caleb B de Lacy, and John G. Purdy. Please ensure that the full contributions of each author are acknowledged in the "Add/Edit/Remove Authors" section of our submission form.

The list of CRediT author contributions may be found here: https://journals.plos.org/plospathogens/s/authorship#loc-author-contributions

2) We ask that a manuscript source file is provided at Revision. Please upload your manuscript file as a .doc, .docx, .rtf or .tex. If you are providing a .tex file, please upload it under the item type u2018LaTeX Source Fileu2019 and leave your .pdf version as the item type u2018Manuscriptu2019.

3) Please upload all main figures as separate Figure files in .tif or .eps format. For more information about how to convert and format your figure files please see our guidelines:

https://journals.plos.org/plospathogens/s/figures

4) We notice that your supplementary Figures are included in the manuscript file. Please remove them and upload them with the file type 'Supporting Information'. Please ensure that each Supporting Information file has a legend listed in the manuscript after the references list.

5) Please note that your Data Availability Statement is currently missing the DOI/accession number of each dataset OR a direct link to access each dataset. If your manuscript is accepted for publication, you will be asked to provide these details on a very short timeline. We therefore suggest that you provide this information now, though we will not hold up the peer review process if you are unable.

6) Please amend your detailed Financial Disclosure statement. This is published with the article. It must therefore be completed in full sentences and contain the exact wording you wish to be published.

1) If the funders had no role in your study, please state: "The funders had no role in study design, data collection and analysis, decision to publish, or preparation of the manuscript."

7) Please ensure that the funders and grant numbers match between the Financial Disclosure field and the Funding Information tab in your submission form. Note that the funders must be provided in the same order in both places as well. Currently, "the BIO5 Institute Postdoctoral Fellowship program" is missing from the Funding Information tab.

8) Please revise your current Competing Interest statement to the standard "The authors have declared that no competing interests exist.".

Note: If the reviewer comments include a recommendation to cite specific previously published works, please review and evaluate these publications to determine whether they are relevant and should be cited. There is no requirement to cite these works unless the editor has indicated otherwise.

**Reviewers' Comments:**

Reviewer's Responses to Questions

**Part I - Summary**

Please use this section to discuss strengths/weaknesses of study, novelty/significance, general execution and scholarship.

Reviewer #1: In this newly submitted manuscript, Mory, et al. interrogate how human cytomegalovirus (HCMV) uses glutamine as a potential carbon source for fatty acid (FA) and lipid biogenesis. Here, the investigators show the metabolic impact is more complicated than previously thought. They demonstrate glutamine contributes directly to lipid synthesis, and does so across lipid tail lengths and degrees of desaturations. Additionally, the authors find HCMV infection results in reductive carboxylation, during which glutamine ultimately is responsible for supplying acetyl-CoA for FA synthesis. The authors have also identified an additional glutamine carbon flow, which is likely facilitated via enzymatic reactions in the malic pathway. Finally, the authors also take a clever approach to return nutrient levels to physiological concentrations. By doing so, they show HCMV promotes FA synthesis and elongation, though physiological levels result in a reduction of viral replication. Overall, the authors’ conclusions are nicely supported by the data. The manuscript is also well-written, and the authors provide helpful schematics throughout to allow the reader to understand the metabolic pathway at-hand. Finally, the overall model presented in figure 9 nicely shows the difference between what we knew (panel A) and what this study now reveals (panel B). Comments for the authors’ consideration are provided below (all minor).

Reviewer #2: This manuscript explores the carbon sources utilized to support lipogenesis during HCMV infection. Using 13C glutamine tracers, they find that glutamine carbon is enriched in various lipids and fatty acids during HCMV infection, indicative of reductive TCA cycle flow. They found that this occurs in supraphysiological as well as more physiologically relevant glucose and glutamine concentrations. The manuscript is very well written, and the lipidomics and tracer experiments are methodologically rigorous. Further, the experiments support their conclusions. However, reductive flow of glutamine to lipids is a widely accepted cellular metabolic activity, with papers describing it over 15 years ago (PMID: 18364355), with subsequent confirmation in many contexts at the time (PMID: 22101433; 22101431). These findings limit the importance of describing these phenotypes in infected cells. The manuscript does not provide new insights into how HCMV might directly modulate specific metabolic activities, nor do they examine how specific metabolic activities might contribute to productive infection. They speculate about contributions that malic enzyme or IDH might be making, but they never test their role. As it stands, their findings are not likely to be of broad general interest and are likely more suitable for a more specialized journal.

**********

**Part II – Major Issues: Key Experiments Required for Acceptance**

Please use this section to detail the key new experiments or modifications of existing experiments that should be absolutely required to validate study conclusions.

Generally, there should be no more than 3 such required experiments or major modifications for a "Major Revision" recommendation. If more than 3 experiments are necessary to validate the study conclusions, then you are encouraged to recommend "Reject".

Reviewer #1: N/A

Reviewer #2: 1. Is malic enzyme important for HCMV-induced lipid biosynthesis or viral infection? What about the activities of IDH isoforms, e.g., IDH1 or IDH2? The authors propose these possibilities, but don’t test the possible contributions to lipid biosynthesis or viral infection.

2. It is not clear whether HCMV infection is increasing the reductive to oxidative ratio of glutamine-carbon into lipid species relative to mock infection. While the authors find increased 13C-glutamine labeling of lipids/fatty acids, given that HCMV dramatically induces lipogenesis during infection, the increased tracer labeling could reflect this increased lipid biosynthesis and not necessarily a change in the oxidative/reductive processing of glutamine carbon (via aKG).

3. They saw a strong reduction in viral titers when comparing rich media to the media with 5 mM glucose & 0.55 mM glutamine (Fig 7F). They indicate that they change the media at 48h post-infection. Given the more than 100-fold effect observed, it raises the question as to whether the media concentration of glucose and glutamine drops precipitously, maybe at 72-120 h post-infection, causing a starvation/autophagic type of response. This is also relevant to whether media concentrations stay at physiologically relevant concentrations, i.e., as opposed to dropping significantly lower.

**********

**Part III – Minor Issues: Editorial and Data Presentation Modifications**

Please use this section for editorial suggestions as well as relatively minor modifications of existing data that would enhance clarity.

Reviewer #1: 1. Define abbreviations at first use (e.g., line 124 – PC, PE, etc).

2. Paragraph ending on line 135 – perhaps a brief phrase detailing what no alteration in PG might suggest is warranted.

3. Line 148 – re: diunsaturated. This looks to only be the case for PC and not PE? Perhaps clarify?

4. Figure 7, figure legend – define LOQ

5. TCID50 data in Figure 7 – what were the media conditions used for the titering assay? Were both the 25/4 and 5/0.55 conditions being assessed normalized into one media for the TCID50 assay, or were they each retained in their respective media for titering? Could this impact the output?

6. Lines 340-1 – since this decrease occurs in mock cells, could this also be a reason for the decrease observed in infected cells between the two conditions (ie: a cell phenotype vs. virus phenotype)?

7. Line 370 – extra word before HCMV? (remove ‘the’?)

Reviewer #2: Minor

1. In Fig 4A, in the schematic, it’s probably worth showing how the labeled 5’ position of glutamine is lost during oxidative TCA cycling.

2. It seems strange that they see 106 viral titers at MOI=1 but 104 at MOI=3.0 (Fig 7F &7G). Is this MOI dependence in replication real/repeatable?

**********

PLOS authors have the option to publish the peer review history of their article (what does this mean?). If published, this will include your full peer review and any attached files.

If you choose “no”, your identity will remain anonymous but your review may still be made public.

**Do you want your identity to be public for this peer review?**  For information about this choice, including consent withdrawal, please see our Privacy Policy.

Reviewer #1: No

Reviewer #2: No

[NOTE: If reviewer comments were submitted as an attachment file, they will be attached to this email and accessible via the submission site. Please log into your account, locate the manuscript record, and check for the action link "View Attachments". If this link does not appear, there are no attachment files.]

**Figure resubmission:**

After uploading your figures to PLOS’s NAAS tool - https://ngplosjournals.pagemajik.ai/artanalysis, NAAS will process the files provided and display the results in the "Uploaded Files" section of the page as the processing is complete. If the uploaded figures meet our requirements (or NAAS is able to fix the files to meet our requirements), the figure will be marked as "fixed" above. If NAAS is unable to fix the files, a red "failed" label will appear above. When NAAS has confirmed that the figure files meet our requirements, please download the file via the download option, and include these NAAS processed figure files when submitting your revised manuscript.

**Reproducibility:**

To enhance the reproducibility of your results, we recommend that authors of applicable studies deposit laboratory protocols in protocols.io, where a protocol can be assigned its own identifier (DOI) such that it can be cited independently in the future. Additionally, PLOS ONE offers an option to publish peer-reviewed clinical study protocols. Read more information on sharing protocols at https://plos.org/protocols?utm_medium=editorial-email&utm_source=authorletters&utm_campaign=protocols

# Author comment: Author response to Decision Letter 1

**DOI:** 10.1371/journal.ppat.1014496.r002

21 Jul 2026

Attachment Submitted filename: Response To Reviewers.docx

# Editor report: Decision Letter 1

**DOI:** 10.1371/journal.ppat.1014496.r003

**Authors:** Eain Murphy

22 Jul 2026

Dear Dr. Purdy,

We are pleased to inform you that your manuscript 'Glutamine supports human cytomegalovirus induced lipidome remodeling through reductive carboxylation' has been provisionally accepted for publication in PLOS Pathogens.

Before your manuscript can be formally accepted you will need to complete some formatting changes, which you will receive in a follow up email. A member of our team will be in touch with a set of requests.

Please note that your manuscript will not be scheduled for publication until you have made the required changes, so a swift response is appreciated.

IMPORTANT: The editorial review process is now complete. PLOS will only permit corrections to spelling, formatting or significant scientific errors from this point onwards. Requests for major changes, or any which affect the scientific understanding of your work, will cause delays to the publication date of your manuscript.

Should you, your institution's press office or the journal office choose to press release your paper, you will automatically be opted out of early publication. We ask that you notify us now if you or your institution is planning to press release the article. All press must be co-ordinated with PLOS.

Thank you again for supporting Open Access publishing; we are looking forward to publishing your work in PLOS Pathogens.

Best regards,

Eain A Murphy, Ph.D.

Academic Editor

PLOS Pathogens

Alison McBride

Section Editor

PLOS Pathogens

Sumita Bhaduri-McIntosh

Editor-in-Chief

PLOS Pathogens

orcid.org/0000-0003-2946-9497

Michael Malim

Editor-in-Chief

PLOS Pathogens

orcid.org/0000-0002-7699-2064

***********************************************************

Dear Dr. Purdy,

Thank you for submitting your revised manuscript to PLoS Pathogens. Based on the additional experimentation and clarification of the text in terms of conclusions and novelty, it is the opinion of the editorial board that this manuscript is now suitable for publication without additional external review. Congratulations on a nice body of work. The manuscript was a pleasure to read.

Sincerely,

Eain Murphy Ph.D.

Reviewer Comments (if any, and for reference):

# Editor report: Acceptance letter

**DOI:** 10.1371/journal.ppat.1014496.r004

**Authors:** Eain Murphy

Dear Dr. Purdy,

We are delighted to inform you that your manuscript, "Glutamine supports human cytomegalovirus induced lipidome remodeling through reductive carboxylation," has been formally accepted for publication in PLOS Pathogens.

We have now passed your article onto the PLOS Production Department who will complete the rest of the pre-publication process. All authors will receive a confirmation email upon publication.

The corresponding author will soon be receiving a typeset proof for review, to ensure errors have not been introduced during production. Please review the PDF proof of your manuscript carefully, as this is the last chance to correct any scientific or type-setting errors. Please note that major changes, or those which affect the scientific understanding of the work, will likely cause delays to the publication date of your manuscript. Note: Proofs for Front Matter articles (Pearls, Reviews, Opinions, etc...) are generated on a different schedule and may not be made available as quickly.

Soon after your final files are uploaded, the early version of your manuscript, if you opted to have an early version of your article, will be published online. The date of the early version will be your article's publication date. The final article will be published to the same URL, and all versions of the paper will be accessible to readers.

For Research Articles, you will receive an invoice from PLOS for your publication fee after your manuscript has reached the completed accept phase. If you receive an email requesting payment before acceptance or for any other service, this may be a phishing scheme. Learn how to identify phishing emails and protect your accounts at https://explore.plos.org/phishing.

Thank you again for supporting open-access publishing; we are looking forward to publishing your work in PLOS Pathogens.

Best regards,

Sumita Bhaduri-McIntosh

Editor-in-Chief

PLOS Pathogens

orcid.org/0000-0003-2946-9497

Michael Malim

Editor-in-Chief

PLOS Pathogens

orcid.org/0000-0002-7699-2064
